# A Structural and Functional Comparison Between Infectious and Non-Infectious Autocatalytic Recombinant PrP Conformers

**DOI:** 10.1371/journal.ppat.1005017

**Published:** 2015-06-30

**Authors:** Geoffrey P. Noble, Daphne W. Wang, Daniel J. Walsh, Justin R. Barone, Michael B. Miller, Koren A. Nishina, Sheng Li, Surachai Supattapone

**Affiliations:** 1 Departments of Biochemistry and Medicine, Geisel School of Medicine at Dartmouth, Hanover, New Hampshire, United States of America; 2 Medicine and Biomedical Sciences Graduate Program, University of California at San Diego, La Jolla, California, United States of America; 3 Department of Biological Systems Engineering, Virginia Tech, Blacksburg, Virginia, United States of America; University of Alberta, CANADA

## Abstract

Infectious prions contain a self-propagating, misfolded conformer of the prion protein termed PrP^Sc^. A critical prediction of the protein-only hypothesis is that autocatalytic PrP^Sc^ molecules should be infectious. However, some autocatalytic recombinant PrP^Sc^ molecules have low or undetectable levels of specific infectivity in bioassays, and the essential determinants of recombinant prion infectivity remain obscure. To identify structural and functional features specifically associated with infectivity, we compared the properties of two autocatalytic recombinant PrP conformers derived from the same original template, which differ by >10^5^-fold in specific infectivity for wild-type mice. Structurally, hydrogen/deuterium exchange mass spectrometry (DXMS) studies revealed that solvent accessibility profiles of infectious and non-infectious autocatalytic recombinant PrP conformers are remarkably similar throughout their protease-resistant cores, except for two domains encompassing residues 91-115 and 144-163. Raman spectroscopy and immunoprecipitation studies confirm that these domains adopt distinct conformations within infectious versus non-infectious autocatalytic recombinant PrP conformers. Functionally, *in vitro* prion propagation experiments show that the non-infectious conformer is unable to seed mouse PrP^C^ substrates containing a glycosylphosphatidylinositol (GPI) anchor, including native PrP^C^. Taken together, these results indicate that having a conformation that can be specifically adopted by post-translationally modified PrP^C^ molecules is an essential determinant of biological infectivity for recombinant prions, and suggest that this ability is associated with discrete features of PrP^Sc^ structure.

## Author Summary

A key prediction of the prion hypothesis is that autocatalytic, misfolded PrP^Sc^ molecules should be highly infectious. Various recombinant PrP^Sc^ conformers are able to self-propagate *in vitro*, yet paradoxically only some of these conformers possess significant levels of specific infectivity in bioassays. Here we use two closely-matched autocatalytic recombinant PrP conformers that share the same origin but differ by >10^5^-fold in specific infectivity to study the molecular basis of prion infectivity. We show that infectious and non-infectious autocatalytic recombinant PrP conformers have subtle structural differences, and that GPI-anchored PrP substrate molecules can only adopt the infectious PrP^Sc^ conformation. We conclude that post-translational modifications of host PrP^C^ molecules play a critical role in restricting the range of recombinant PrP^Sc^ conformers that are biologically infectious.

## Introduction

The conformational conversion of the host-encoded prion protein (PrP) is a central pathogenic event in the prion diseases [1]. In healthy individuals, PrP adopts a fold that is rich in α-helix, termed PrP^C^, and is post-translationally modified by the incorporation of N-linked glycans and a C-terminal glycosylphosphatidylinositol (GPI) anchor. In individuals suffering from prion disease, PrP^C^ is misfolded into a β-sheet rich conformation, termed PrP^Sc^, which is capable of acting as a template for the conformational conversion of additional PrP^C^ molecules into PrP^Sc^. This self-propagating activity of PrP^Sc^ is referred to as autocatalysis and is thought to underlie the infectious nature of the prion diseases. A critical prediction of the protein-only hypothesis is that autocatalytic PrP^Sc^ molecules should be infectious.

A number of *in vitro* techniques for generating misfolded, autocatalytic PrP^Sc^ conformers have been developed and refined, including the cell-free conversion assay [2] and the serial protein misfolding cyclic amplification (sPMCA) technique [3,4]. With few exceptions, PrP^Sc^ conformers derived from post-translationally modified, native PrP^C^ substrates have been highly infectious when bioassayed in wild-type animals [4–8]. In contrast, various autocatalytic PrP^Sc^ conformers derived from recombinant PrP substrates lacking post-translational modifications have displayed large variations in specific infectivity levels as determined by bioassay in wild-type animals [9–15]. The structural and functional basis of this striking variability in specific infectivity between different autocatalytic recombinant PrP^Sc^ molecules remains unknown.

Recently, using only bacterially expressed recombinant PrP and a single endogenous phospholipid cofactor molecule, phosphatidylethanolamine (PE), as substrates, Deleault *et al*. successfully produced high titer (2.2 x 10^6^ LD_50_ U/μg PrP), chemically defined mouse prions *in vitro* [10]. Interestingly, it was observed that the prions produced from these minimal components always formed into a single infectious strain with unique, novel biological properties regardless of the seed originally used to template the *in vitro* reactions. Importantly, when this novel prion strain was subsequently propagated in the absence of PE cofactor, a new misfolded recombinant PrP conformer was produced, which could also self-propagate in sPMCA reactions, but which surprisingly failed to cause disease upon injection into wild-type mice [10]. This new autocatalytic conformer, which we refer to as protein-only PrP^Sc^, therefore had a >10^5^-fold lower level of specific infectivity as compared to the PrP^Sc^ conformer produced in the presence of PE cofactor, which we refer to as cofactor PrP^Sc^.

We saw an opportunity to identify structural and functional properties associated with recombinant PrP^Sc^ infectivity by directly comparing these two related PrP^Sc^ conformers, which share the same origin and autocatalytic behavior, but differ strikingly in biological infectivity.

## Results

### Functional differences between cofactor and protein-only PrP^Sc^ can be localized to their respective C-terminal, protease-resistant cores

**Fig 1:**
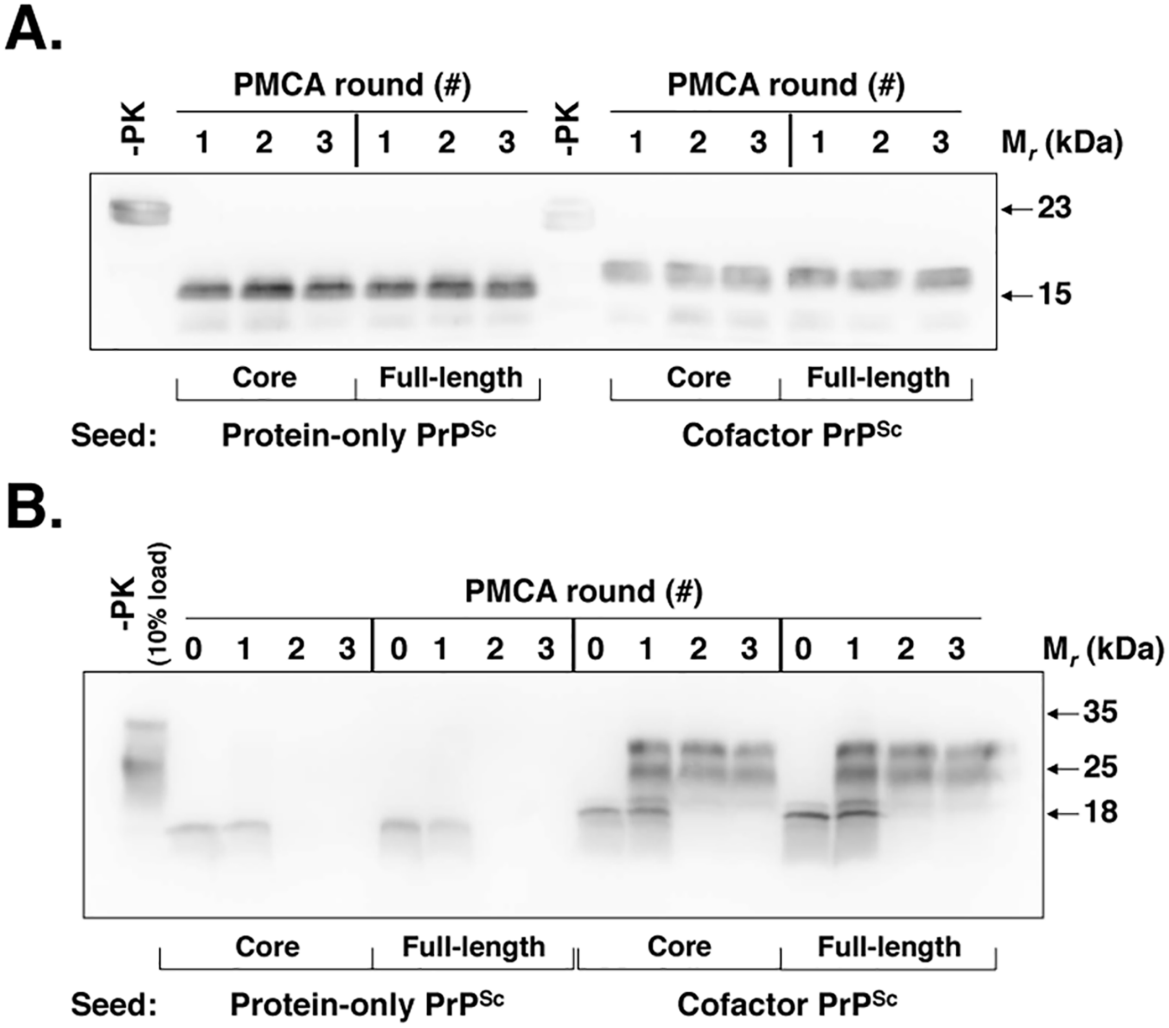
Cofactor and protein-only PrP^Sc^ are stably propagating recombinant PrP conformers that differ in their ability to template the conversion of native PrP^C^. (*A*) Western blot showing three-round sPMCA reactions using recombinant PrP as the substrate and seeded with full-length or PK-digested cofactor and protein-only PrP^Sc^, as indicated. (*B*) Western blot showing three-round sPMCA reactions using normal mouse brain homogenate as the substrate and seeded with full-length or PK-digested cofactor and protein-only PrP^Sc^, as indicated.

Cofactor and protein-only PrP^Sc^ are distinct misfolded recombinant PrP conformers that differ >10^5^-fold in their specific infectivity for wild-type mice [10]. While both of these conformers demonstrate autocatalytic activity when used to seed sPMCA reactions containing recombinant PrP substrate (Fig 1A and [10]), only cofactor PrP^Sc^ also demonstrates autocatalysis when used to seed sPMCA reactions containing normal brain homogenate as the substrate (Fig 1B and [10]). The complete failure of protein-only PrP^Sc^ to function as a seed for conversion reactions containing native PrP^C^ substrate (Fig 1B, left sample group) provides a logical explanation for this conformer’s lack of infectious activity *in vivo*, and may apply more generally to other recombinant PrP^Sc^ conformers which demonstrate low levels of specific infectivity in bioassays. Using cofactor PrP^Sc^ as a well-matched control, we therefore sought to gain structural and mechanistic insight into the substrate-dependence of protein-only PrP^Sc^ autocatalytic activity as a means to understand the structural and functional determinants of recombinant PrP^Sc^ infectivity.

To help focus the structural comparison between cofactor and protein-only PrP^Sc^ molecules, we first tested whether the protease-resistant cores of both conformers, which contain approximately two thirds of the residues of mature full-length PrP, have the same substrate-specific activity as their respective parent PrP^Sc^ molecules in *in vitro* propagation experiments (Fig 1). We found that in sPMCA reactions containing recombinant PrP substrate and defined cofactors, both full-length and truncated cofactor and protein-only PrP^Sc^ molecules function as competent seeds which faithfully propagate the characteristic PK-resistant bands associated with their parent PrP^Sc^ molecules (Fig 1A) [10]. Moreover, in *in vitro* conversion reactions containing native, brain-derived PrP^C^ substrate we found that full-length and PK-digested cofactor PrP^Sc^ drive the conversion of native PrP^C^ (Fig 1B, third and fourth panels), while full-length and truncated protein-only PrP^Sc^ do not (Fig 1B, first and second panels), indicating that functional differences in the *in vitro* activity of cofactor and protein-only PrP^Sc^ molecules can be localized to the PK-resistant core. Epitope mapping of the cofactor and protein-only PrP^Sc^ PK-resistant cores (S1 Fig), revealed that both are C-terminal PrP fragments that include the 6D11 epitope (residues 93–109, with 97–100 as the major determinants of binding [16]) (S1 Fig, top panel) and the extreme C-terminus (S1 Fig, bottom panel). Based on these results, we focused our subsequent structural analyses on C-terminal residues beginning at glycine 89 (G89), the primary PK-cleavage site for PrP^Sc^ 27–30 [17].

### Structural comparison of cofactor and protein-only PrP^Sc^ by DXMS identifies conformational differences in restricted C-terminal domains

**Fig 2:**
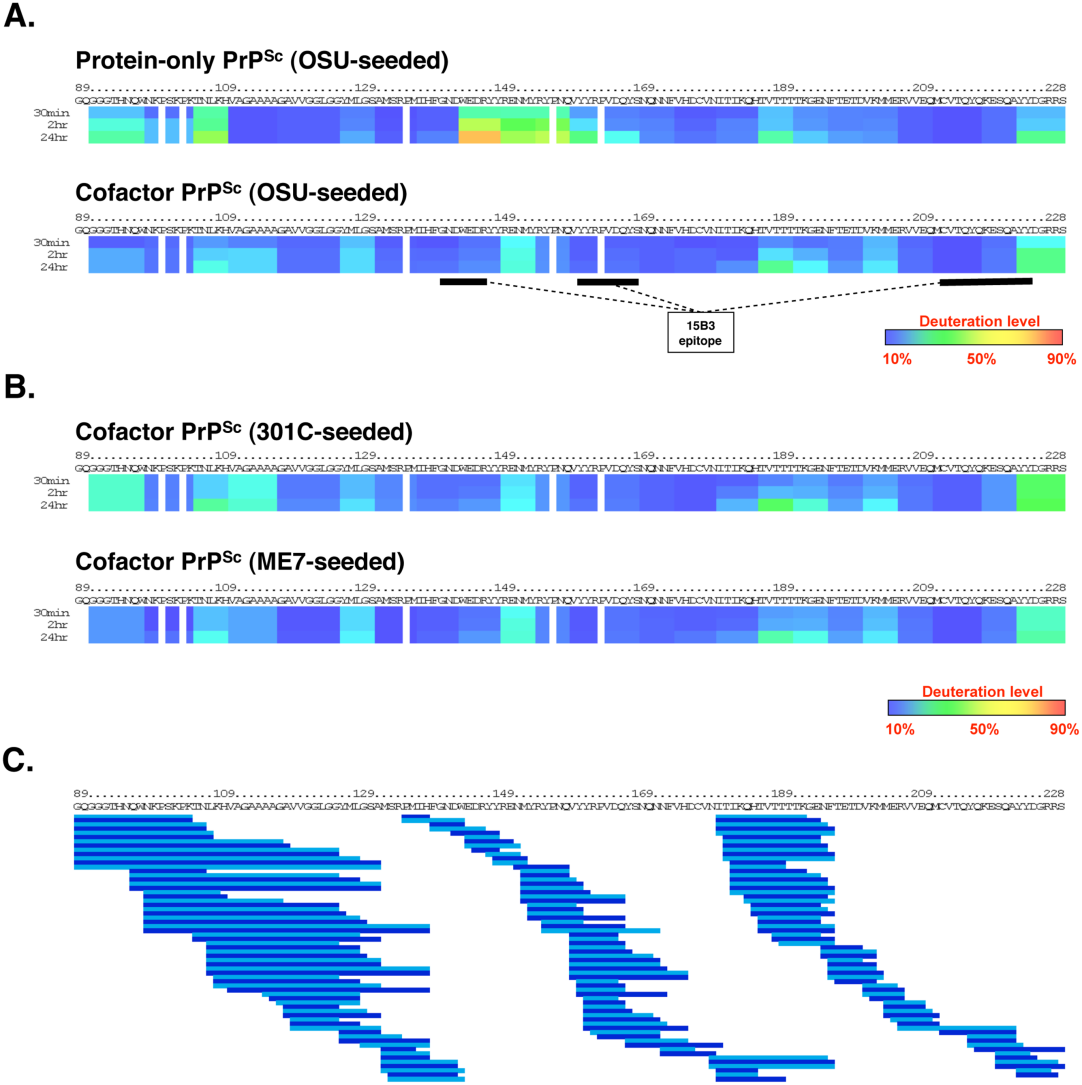
Regional solvent accessibility of cofactor and protein-only PrP^Sc^ conformers. Purified PrP^Sc^ conformers were incubated in D_2_O-containing exchange buffer for 30 min, 2 h, or 24 h and the reaction products were quenched and subjected to LC/MS to measure peptide-specific deuterium incorporation. Data from overlapping peptides was used to quantify localized deuterium exchange and construct ribbon diagrams from a representative experiment. (*A*) Regional solvent accessibility comparison between cofactor and protein-only PrP^Sc^ conformers derived from the same OSU prion strain. The discontinuous epitope of mAb 15B3, which selectively binds infectious PrP^Sc^, is indicated with black bars [18]. (*B*) Regional solvent accessibility of two additional cofactor PrP^Sc^ conformers, derived from the 301C and ME7 prion strains. (*C*) Map of the 188 high quality deuterated peptides, including different peptide charge states, identified in all four PrP^Sc^ experimental samples and used to construct ribbon diagrams in parts (*A*) and (*B*). Alternating shades of blue in part (*C*) are used to highlight neighboring peptides. Despite complete coverage of amino acids 89–230 by overlapping peptides, gaps exist in the ribbon diagrams shown in parts (*A*) and (*B*) for two reasons: 1. Deuterium incorporation cannot be quantified for the two N-terminal residues of a peptide as a result of rapid back exchange [19], and 2. Proline residues lack amide hydrogen atoms and therefore do not contribute to deuterium incorporation measurements.

To compare the structures of cofactor PrP^Sc^ and protein-only PrP^Sc^ molecules, we performed hydrogen/deuterium exchange MS (DXMS) on these two conformers generated in parallel from the same OSU prion strain seed (Fig 2A). The OSU prion strain was originally synthesized by Wang *et al*. using recombinant PrP, total liver RNA and 1-palmitoyl-2-oleoyl-sn-glycero-3-phosphoglycerol (POPG) [15], and it has previously been referred to as the OSU strain by Deleault *et al*. [10]. OSU-seeded cofactor and protein-only PrP^Sc^ samples used for structural analysis in this study were generated with high conversion efficiency in sPMCA (S2 Fig) and then purified prior to DXMS analysis by a series of ultracentrifugation steps described in Materials and Methods. Co-sedimentation of significant quantities of non-specifically aggregated, protease-sensitive PrP was ruled out in the OSU-seeded cofactor PrP^Sc^ DXMS sample due to its near complete conversion to PK-resistant PrP^Sc^ (96% PK-resistant conversion efficiency, S2 Fig). To assess the potential contribution of non-specifically aggregated, protease-sensitive PrP to the OSU-seeded protein-only PrP^Sc^ sample analyzed by DXMS (77% PK-resistant conversion efficiency, S2 Fig), we mock-seeded protein-only PMCA reactions and subjected the resulting material to the DXMS purification protocol (S3 Fig, top panel). We did not detect any non-specifically aggregated PrP in these mock-seeded PMCA reactions (S3 Fig, top panel, samples S_0_ vs P_3_), indicating that the protein-only PrP^Sc^ DXMS sample does not contain appreciable quantities of non-specifically aggregated, protease sensitive PrP.

Regional solvent accessibility of cofactor and protein-only PrP^Sc^ was determined by incorporating deuteration data from 188 overlapping C-terminal peptides (Fig 2C) recovered in a representative experiment from each deuterium-labeled PrP^Sc^ sample. This high density of overlapping deuterated peptides provides PrP^Sc^ solvent accessibility measurements with resolution down to segments of ~5 amino acids. In examining and discussing the results of this study, specific regions of the PrP primary sequence are referred to either by explicit residue numbering, based on the mouse PrP sequence, or with reference to the location of known secondary structural elements in monomeric, α-helical recombinant PrP (α-PrP). For example, the domain corresponding to residues 178–216 could also be described as α_2_-α_3_ because it encompasses the second and third α-helices in α-PrP.

The C-terminal cores of cofactor and protein-only PrP^Sc^ are both substantially protected from solvent exchange (Fig 2A) as compared to α-PrP (S4 Fig). This solvent protection is consistent with widespread conversion to β-sheet secondary structure and/or the formation of large solvent-excluding aggregates [20]. Within the misfolded, solvent-protected PrP^Sc^ core there are large regions in which cofactor PrP^Sc^ and protein-only PrP^Sc^ have remarkably similar solvent accessibility profiles—in particular, the regions containing residues 118–143 and 165–230. However, there are also specific domains in which the cofactor and protein-only conformations can be distinguished by solvent accessibility. Most clearly, the domain encompassing residues 144–163, corresponding to α_1_ and β_2_ in α-PrP [21], is more solvent-exposed in protein-only PrP^Sc^ than in cofactor PrP^Sc^ (Fig 2A). The relatively exposed structure of the α_1_-β_2_ domain was preserved, and in fact accentuated, in an independently prepared protein-only PrP^Sc^ sample (S5 Fig). In addition, residues 91–110 appear to be slightly more exposed in protein-only PrP^Sc^ than in cofactor PrP^Sc^ while residues in the palindromic region (amino acids 111–120) are more obviously protected in protein-only PrP^Sc^. Differences in these relatively N-terminal regions may contribute to the differing susceptibility to PK cleavage observed for the cofactor and protein-only PrP^Sc^ conformations (Fig 1 and S1 and S2 Figs).

### Convergence of biological strain properties is correlated with cofactor PrP^Sc^ structural convergence

Deleault *et al*. [10] originally used three prion strains with distinct infectious phenotypes to seed the chemically-defined, recombinant PrP conversion system used to produce cofactor and protein-only PrP^Sc^ molecules. Interestingly, the three input strains converged into a single strain with a novel biological phenotype upon propagation in sPMCA reactions containing only recombinant PrP substrate and a single cofactor. We used DXMS to examine the structures of the two additional cofactor PrP^Sc^ samples generated by Deleault *et al*. in sPMCA reactions that were initially seeded with mouse prion strains distinct from the OSU strain (301C- and ME7-seeded cofactor PrP^Sc^) (Fig 2B). The results revealed that the PK-resistant cores of all three cofactor PrP^Sc^ molecules have nearly identical solvent accessibility profiles (Fig 2A and 2B), consistent with convergence into a single cofactor PrP^Sc^ conformation. As was the case with OSU-seeded cofactor and protein-only PrP^Sc^, we assessed the contribution of non-specifically aggregated, protease-sensitive PrP to the DXMS data for 301C- and ME7-seeded cofactor PrP^Sc^ by determining sample conversion efficiency (S2 Fig) and performing a mock-seeding experiment (S3 Fig, bottom panel). 301C-seeded cofactor PrP^Sc^ is almost entirely converted to PK-resistant PrP^Sc^ (99% PK-resistant conversion efficiency, S2 Fig), ruling out any significant contribution of non-specific PrP aggregation to the presented DXMS data. Mock-seeding of cofactor-supplemented PMCA reactions resulted in the recovery of approximately 8% of the starting material as non-specifically aggregated, protease-sensitive PrP (S3 Fig, bottom panel, samples S_0_ vs P_3_). For ME7-seeded cofactor PrP^Sc^ (82% PK-resistant conversion efficiency, S2 Fig), we therefore estimate that such non-specifically aggregated PrP accounts for no more than 2% of the sample analyzed by DXMS (ie. 8% of the unconverted, PK-sensitive material yields the estimate for non-specifically aggregated, PK-sensitive PrP, or ~1.4% of the total input PrP, which is then divided by the sum of the PK-resistant and PK-sensitive insoluble PrP, or ~83.4% of the total input PrP).

The data from cofactor PrP^Sc^ and protein-only PrP^Sc^ sample replicates were aggregated, yielding 63 shared peptides for which individual deuteration curves could be plotted (S6 and S7 Figs). Interestingly, peptides that include residues N-terminal to G89 were less frequently recovered and showed irregular deuteration profiles specifically in cofactor PrP^Sc^ samples (S6 and S7 Figs).

### Domain-specific conformational differences between cofactor and protein-only PrP^Sc^ are supported by immunoprecipitation and Raman spectroscopy studies

**Fig 3:**
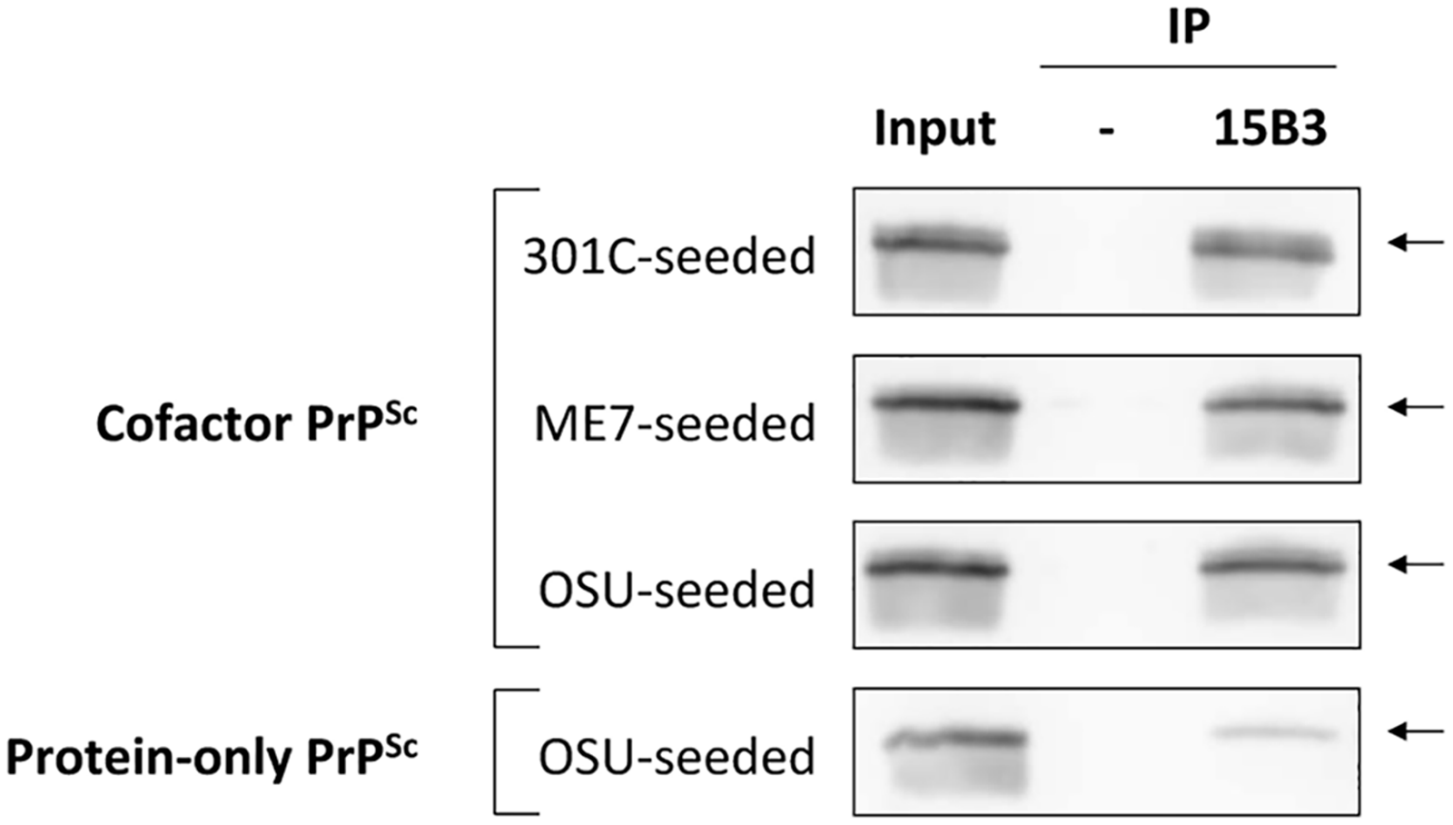
Immunoprecipitation with conformation-specific mAb 15B3 distinguishes between cofactor and protein-only PrP^Sc^. Converted sPMCA products were purified by ultracentrifugation with nOG washes to remove unconverted α-PrP and excess lipid, and immunoprecipation was performed using 15B3-coated or uncoated rat anti-mouse IgM-conjugated magnetic beads, as indicated. The location of the discontinuous, 15B3 conformational epitope is shown in Fig 2A. Arrows indicate an M_r_ of ~23 kDa, the expected mobility of full-length recombinant PrP. By densitometry, the efficiency of 15B3 immunoprecipitation in this experiment is 79%, 71% and 74% for 301C-seeded, ME7-seeded, and OSU-seeded cofactor PrP^Sc^, respectively, and 15% for OSU-seeded protein-only PrP^Sc^.

Having identified the α_1_-β_2_ domain by DXMS as a region of conformational divergence in our cofactor and protein-only PrP^Sc^ samples, we sought to confirm this finding using additional biochemical and biophysical approaches. The α_1_-β_2_ region contains a portion of the epitope for 15B3, a well-characterized PrP^Sc^-specific conformational antibody [18]. The regions of PrP primary structure that comprise the discontinuous 15B3 epitope are shown schematically in Fig 2A. In a single immunoprecipitation experiment, 15B3 efficiently pulled down all three of our cofactor PrP^Sc^ samples (Fig 3, top three panels), but only weakly bound protein-only PrP^Sc^ (Fig 3, bottom panel), indicating a disruption of the 15B3 conformational epitope, consistent with our DXMS results.

**Fig 4:**
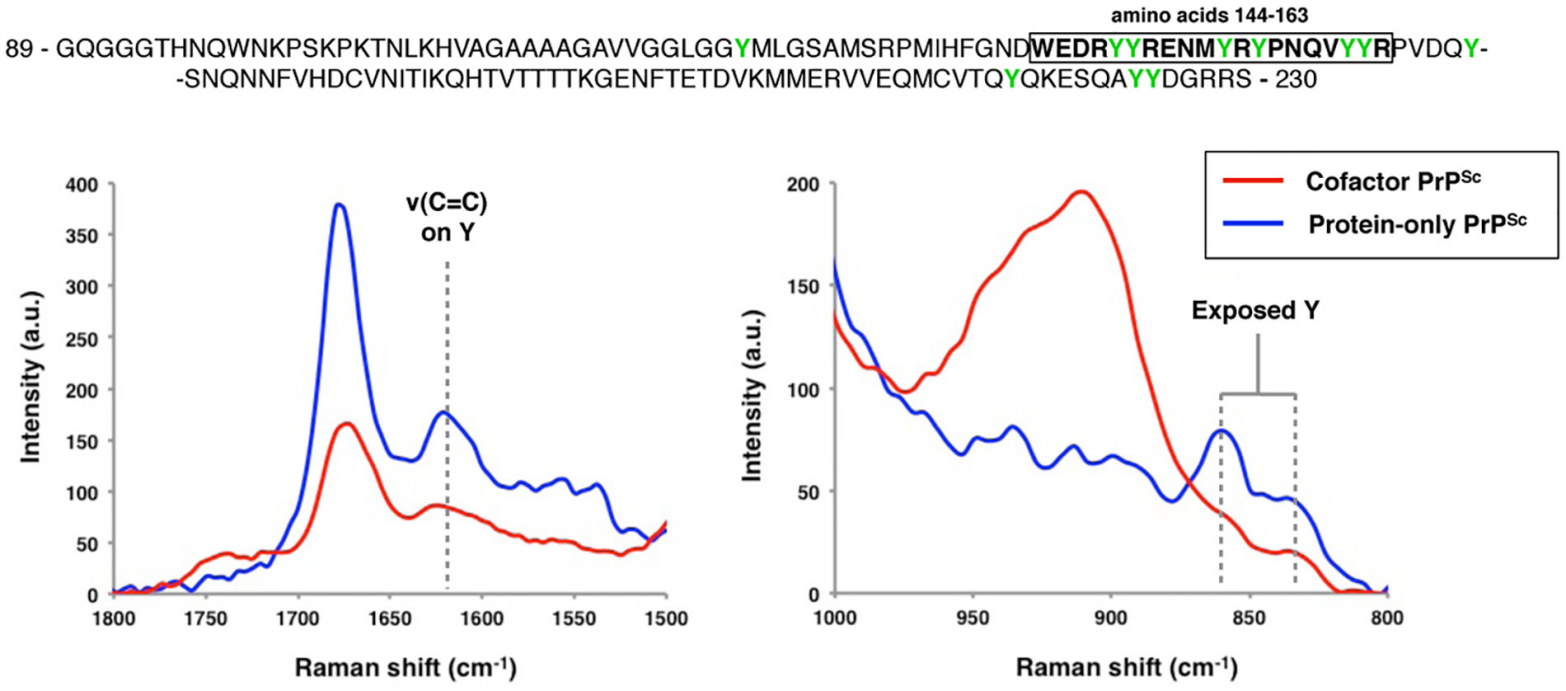
Raman spectroscopy of cofactor and protein-only PrP^Sc^, focusing on spectral regions assigned to tyrosine side chains. The primary sequence of the region examined by DXMS is shown, with residues 144–163 boxed and tyrosine residues highlighted in green. Raman shifts corresponding to the ν(C = C) ring mode (~1620 cm^-1^) and the tyrosine Fermi-doublet (~850 and 830 cm^-1^) are shown.

We further sought to confirm a conformational difference between cofactor and protein-only PrP^Sc^ in the α_1_-β_2_ domain using Raman spectroscopy (Fig 4). Analysis of the Raman spectra acquired from these two conformers identified multiple Raman shifts that could be assigned to tyrosine residues, which are plentiful in the PrP C-terminus and specifically enriched in the α_1_-β_2_ domain (6 of 11 total C-terminal tyrosines). By Raman spectroscopy, protein-only PrP^Sc^ appears to contain more exposed tyrosine residues than cofactor PrP^Sc^ as evidenced from the increased ring ν(C = C) intensity at ~1620 cm^-1^ (Fig 4, left panel), the 850 cm^-1^/830 cm^-1^ ratio being greater than 1 (Fig 4, right panel) [22], and the increased ring ν(CH) intensity at ~3075 cm^-1^ (S8 Fig, left panel) [23,24], consistent with our DXMS results. In addition, and also consistent with our DXMS data, protein-only PrP^Sc^ appears to contain more exposed CNH groups than cofactor PrP^Sc^ as indicated by the increased intensity in the 1530–1580 cm^-1^ Amide II region, corresponding to ν(CN) and δ(CNH) Raman shifts (S8 Fig, right panel), as well as an increased ν(CN) intensity at ~3300 cm^-1^ (S8 Fig, left panel). These exposed CNH groups likely originate from the 4 exposed arginine (R) and single exposed glutamine (Q), asparagine (N) and tryptophan (W) residues in the α_1_-β_2_ domain, or from CNH-containing side chains in the N-terminal portion of the PK-resistant PrP^Sc^ core (residues ~91–115).

### Cofactor and protein-only PrP^Sc^ differ functionally in the ability to convert GPI-anchored substrates

**Fig 5:**
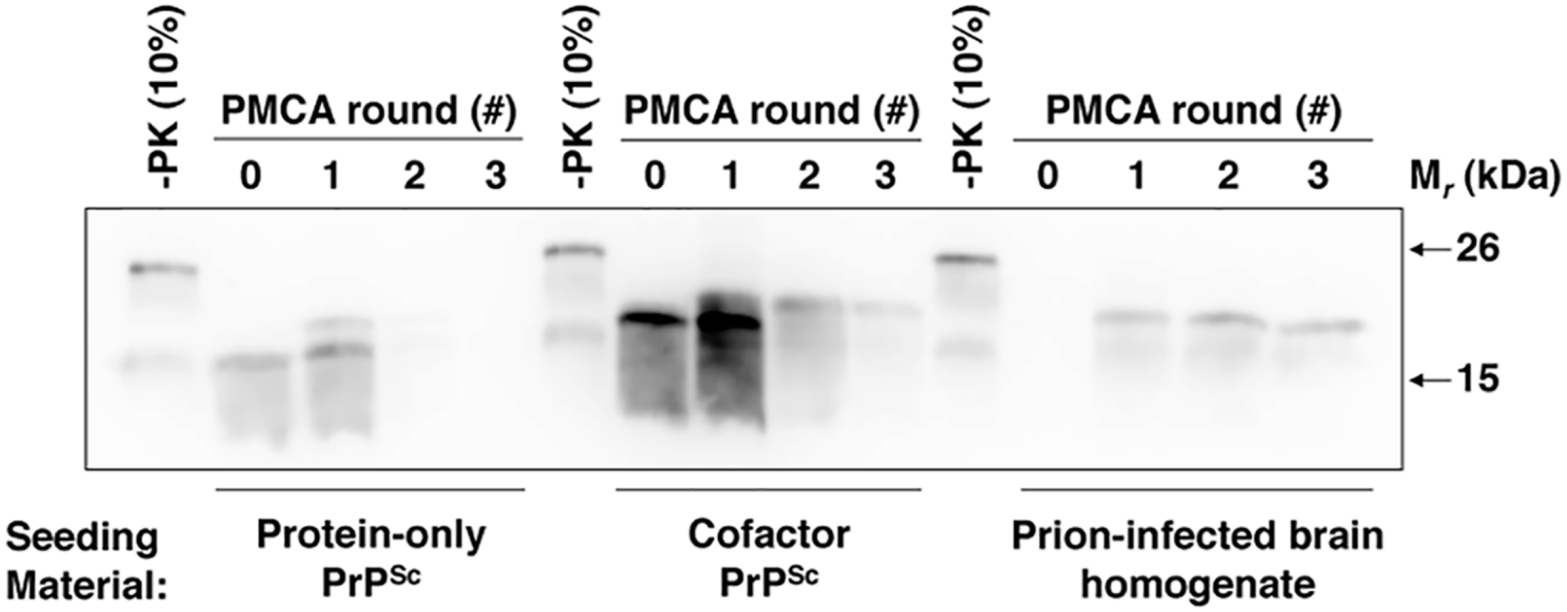
Cofactor and protein-only PrP^Sc^ differ in their ability to template the conversion of PrP substrates containing a GPI anchor. Western blot showing three round sPMCA reactions using partially purified and deglycosylated PrP^C^ as the substrate and seeded with protein-only PrP^Sc^, cofactor PrP^Sc^, or prion-infected brain homogenate, as indicated. A replicate of this experiment is shown in S9 Fig

Our DXMS data suggests that structural differences between cofactor and protein-only PrP^Sc^ are limited to specific domains (Fig 2A) and that these structural differences affect the ability of recombinant PrP^Sc^ to convert native PrP^C^ (Fig 1B). As conversion substrates, α-PrP and native PrP^C^ share the same primary sequence, but PrP^C^ also contains bulky N-linked glycans and a GPI anchor as post-translational modifications. Therefore, we hypothesized that limited conformational differences might dramatically alter a recombinant conformer’s infectious activity by impinging on spatial regions that would be occupied by N-linked glycans or a GPI anchor should PrP^C^ adopt the same conformation. To test this hypothesis, we partially purified native PrP^C^ and performed enzymatic deglycosylation with PNGase F. The resulting PrP^C^ molecules, which uniformly contain a GPI anchor as the sole post-translational modification, were used as the substrate in sPMCA experiments seeded with cofactor and protein-only PrP^Sc^ (Fig 5 and repeated in S9 Fig). Like brain-derived prions, recombinant cofactor PrP^Sc^ was able to template the conversion of unglycosylated PrP^C^ (Fig 5 and S9 Fig, middle and right sample groups), whereas protein-only PrP^Sc^ failed to function as an autocatalytic seed in the same substrate (Fig 5 and S9 Fig, left sample group). Note that initial conversion of unglycosylated PrP^C^ substrate molecules to a PK-resistant form was detected after seeding with both cofactor and protein-only PrP^Sc^ and 24 h of intermittent sonication, as indicated by the appearance of PK digestion products of slightly higher apparent molecular weight than the respective input seeds (Fig 5 and S9 Fig, left and middle sample groups, round 1 vs 0). In the case of protein-only PrP^Sc^, this higher molecular weight product is diluted out in proportion to the initial seed (Fig 5 and S9 Fig, left sample group, round 2 vs 1), suggesting stoichiometric, as opposed to autocatalytic, PrP conversion. In contrast, the higher molecular weight band generated from infectious PrP^Sc^ seed was able to propagate independently of the initial PrP^Sc^ seed, suggesting autocatalytic PrP conversion (Fig 5 and S9 Fig, left and middle sample groups, rounds 1–3). This result indicates that the existence of bulky N-linked substrate glycans is not solely responsible for the failure of recombinant protein-only PrP^Sc^ to function as a seed for native PrP^C^. Moreover, by isolating the effect of a single substrate post-translational modification on recombinant PrP^Sc^ function, this result provides proof of principle that such modifications may impair the efficient replication of certain recombinant PrP^Sc^ conformers *in vivo*.

## Discussion

A misfolded conformer of the prion protein, PrP^Sc^, is an essential, and possibly the sole, component of infectious prions [1]. Although PrP^Sc^ is known to be rich in β-sheet [25–27], a high-resolution structure of this PrP conformer is lacking, and the structural features of PrP^Sc^ that determine its infectious activity remain obscure.

Recently, Deleault *et al*. used a chemically-defined, minimal PrP conversion system and identical seeding material to generate two distinct, autocatalytic recombinant PrP^Sc^ conformers that differ >10^5^-fold in their specific infectivity [10]. One recombinant conformer, produced in the presence of PE cofactor molecules and termed cofactor PrP^Sc^, had a titer in normal C57BL mice nearly equivalent to that of brain-derived PrP^Sc^ (2.2 x 10^6^ LD_50_ U/μg PrP), while the other conformer, produced in the absence of cofactor molecules and termed protein-only PrP^Sc^, failed to cause disease in the same host, even at the most concentrated dose tested. In the present study, we have taken advantage of the uniquely controlled opportunity presented by these two PrP^Sc^ conformers, which share the same origin and autocatalytic behavior but differ strikingly in their biological activity, in order to investigate the structural and functional determinants of recombinant PrP^Sc^ infectivity.

By comparing the structures of these two conformers using DXMS, we find that cofactor and protein-only PrP^Sc^ molecules have remarkably similar solvent accessibility profiles within the PK-resistant PrP^Sc^ core (Fig 2A). Indeed, they are virtually indistinguishable by this measure in the domains that correspond roughly to α_2_-α_3_ in α-helical PrP (~ residues 165–230), and to the stretch of residues between the hydrophobic domain and the start of α_1_ (~118–143) (Fig 2A), both displaying significant protection from solvent exchange, consistent with widespread conversion to β-sheet secondary structure. The degree of solvent protection, especially N-terminal to β_2_, distinguishes cofactor and protein-only PrP^Sc^ from synthetic PrP amyloids [27–31], and is most consistent with previous DXMS studies using brain-derived and recombinant prions [27,32,33]. Like these recent DXMS studies, the data from the present study are consistent only with those models of PrP^Sc^ structure that involve a complete refolding of the PrP^C^ C-terminal α-helices to β-sheet—for example, the parallel in-register β-sheet architectures proposed by Cobb *et al*. [34] and more recently by Groveman *et al*.[35]. Interestingly, the modest increase in solvent accessibility seen at residues 187–196 in all four PrP^Sc^ conformers studied here (Fig 2) corresponds well with the proposed loop of the native disulfide hairpin predicted by both of these models.

In addition to broad similarities between cofactor and protein-only PrP^Sc^ in solvent accessibility, we have identified two specific domains in which the cofactor and protein-only PrP^Sc^ conformers can be conformationally distinguished: most clearly within the domain that corresponds to α_1_ through the C-terminus of β_2_ in PrP^C^ (~ residues 144–163), but also within a domain at the N-terminus of the PK-reistant core, comprising residues ~91–115 (Fig 2A). In both of these domains, protein-only PrP^Sc^ appears to be more exposed to solvent exchange than cofactor PrP^Sc^. Given the role PE cofactor molecules play in the formation of cofactor, but not protein-only, PrP^Sc^ [10], it is likely that the relatively solvent-protected structural features selectively associated with cofactor PrP^Sc^ are cofactor-induced. Moreover, the fact that the PK-resistant cores of all three cofactor PrP^Sc^ samples, derived from distinct prion strains but propagated in the same chemically-defined system, are highly similar (Fig 2A and 2B) suggests that the convergence of biological strain properties observed by Deleault *et al*. [10] is associated with a convergence of PrP^Sc^ structure.

The α_1_-β_2_ domain, which appears to adopt different conformational states in cofactor and protein-only PrP^Sc^ (Figs 2–4), has previously been identified as a region of PrP that has important implications for PrP misfolding. For example, several PrP^Sc^-selective conformational antibodies are known to have epitopes that reside within this domain [18,36], and small deletions towards the C-terminus of this domain produce PrP molecules that do not readily form PrP^Sc^ and, in fact, function as dominant-negative inhibitors of PrP^Sc^ replication in full-length PrP substrates [37]. Moreover, in the complete absence of the α_1_-β_2_ domain, a redacted ‘miniprion’ is capable misfolding to form PrP^Sc^, but does not cause disease when inoculated into animals expressing full-length PrP [38,39]. Interestingly, the α_1_-β_2_ domain lies adjacent the β_2_-α_2_ loop, a region of PrP known to play an important role in prion formation and interspecies prion transmission [40–43].

Less is known about the second domain (amino acids ~91–115) in which cofactor and protein-only PrP^Sc^ appear to adopt different conformational states (Fig 2 and S5 Fig). This domain includes the so-called ‘fifth site’ for Cu^2+^ binding [44–46].

It is important to acknowledge that any comparisons drawn between DXMS results in the present study and the activities of cofactor and protein-only PrP^Sc^ are correlations only, and that it is possible the structural features underlying PrP^Sc^-associated activities such as autocatalysis and infectivity may be subtle and/or beyond the resolution of the DXMS approach. Similarly, it should be acknowledged that DXMS provides a measure of the average deuterium incorporation at a given amide proton position over a population of PrP^Sc^ molecules. It has been proposed that PrP^Sc^ exists as a heterogeneous conformational mixture of so-called quasi-species [47], but it is not known—for the recombinant PrP^Sc^ conformers studied here, or any other PrP^Sc^ preparation—what proportion of PrP^Sc^ molecules exhibit autocatalytic or infectious activity. Therefore, the solvent accessibility profiles obtained in this study are representative of conformational differences at a PrP^Sc^ population level, and may not be representative of rare, and potentially biochemically/biologically active, components within a given PrP^Sc^ sample. In addition, it should be made clear that when interpreting ribbon diagrams, as in Fig 2, similar solvent accessibility profiles do not guarantee similar tertiary and/or quaternary structures, although by using a set of matched peptides with dense, overlapping coverage of the region of interest (Fig 2C) to compare the solvent accessibility of the cofactor and protein-only PrP^Sc^ conformers, we have increased confidence in such an interpretation of the data. Finally, we do not claim that a relatively solvent inaccessible conformation of either of the two structurally divergent domains identified in the present study is required for PrP^Sc^ infectivity generally. The data presented here are specific to the situation in which seed and substrate are both of the mouse PrP sequence, and it is possible that different PrP sequences may have different infectivity-associated conformational spaces. Consistent with a sequence-specific interpretation of our results, it has previously been shown that native mouse prions also appear to have a relatively solvent-protected structure in the α_1_-β_2_ domain [27], while a recent study showed that this same domain is relatively solvent exposed in native human prions [33].

The dissociation of *in vitro* autocatalytic activity and infectivity seen in some, but not all, recombinant PrP^Sc^ conformers [10,14,15] presents an interesting functional question: why is it that a PrP^Sc^ conformer capable of self-replication in recombinant PrP substrate fails to function as a template for native PrP^C^ conversion *in vivo*? One obvious possibility relates to substrate complexity: although α-PrP and native PrP^C^ share similar secondary structures [21,48], native PrP^C^ is a more complex conversion substrate due to the post-translational addition of N-linked glycans and a GPI anchor, and due its location within the membrane environment of cells. Using our cofactor and protein-only recombinant PrP^Sc^ seeds as a controlled pair, we performed *in vitro* conversion experiments in which we, in a step-wise manner, modified native PrP^C^ substrate to make it more and more like α-PrP in an effort to identify the factor(s) that prevent protein-only PrP^Sc^ from converting PrP^C^ *in vivo*. Remarkably, the functional difference between cofactor and protein-only PrP^Sc^ persisted even after extracting native PrP^C^ from the membrane environment (Fig 1B and [10]) and removing all N-linked glycans (Fig 5 and S9 Fig), suggesting that neither of these factors is responsible for preventing protein-only PrP^Sc^ from converting native PrP^C^ *in vivo*. Unfortunately, the complementary experiment, in which the GPI anchor of PrP^C^ is selectively removed and that modified PrP^C^ substrate used in conversion reactions is not possible for two reasons: 1. Delipidated PrP^C^ is a poor conversion substrate, even for sPMCA experiments seeded with native prions [49], and 2. Detection of delipidated GPI-anchored proteins by Western blotting is technically challenging [50]. Nevertheless, we infer from the available data that the functional difference between cofactor and protein-only PrP^Sc^ is mostly likely attributable to differing abilities of these two conformers to template the conversion of wild-type mouse PrP substrates containing a GPI anchor. Interestingly, Kim *et al*. have previously demonstrated that the GPI anchor plays an important role in the *in vitro* formation of native PrP^Sc^ conformers [49].

**Fig 6:**
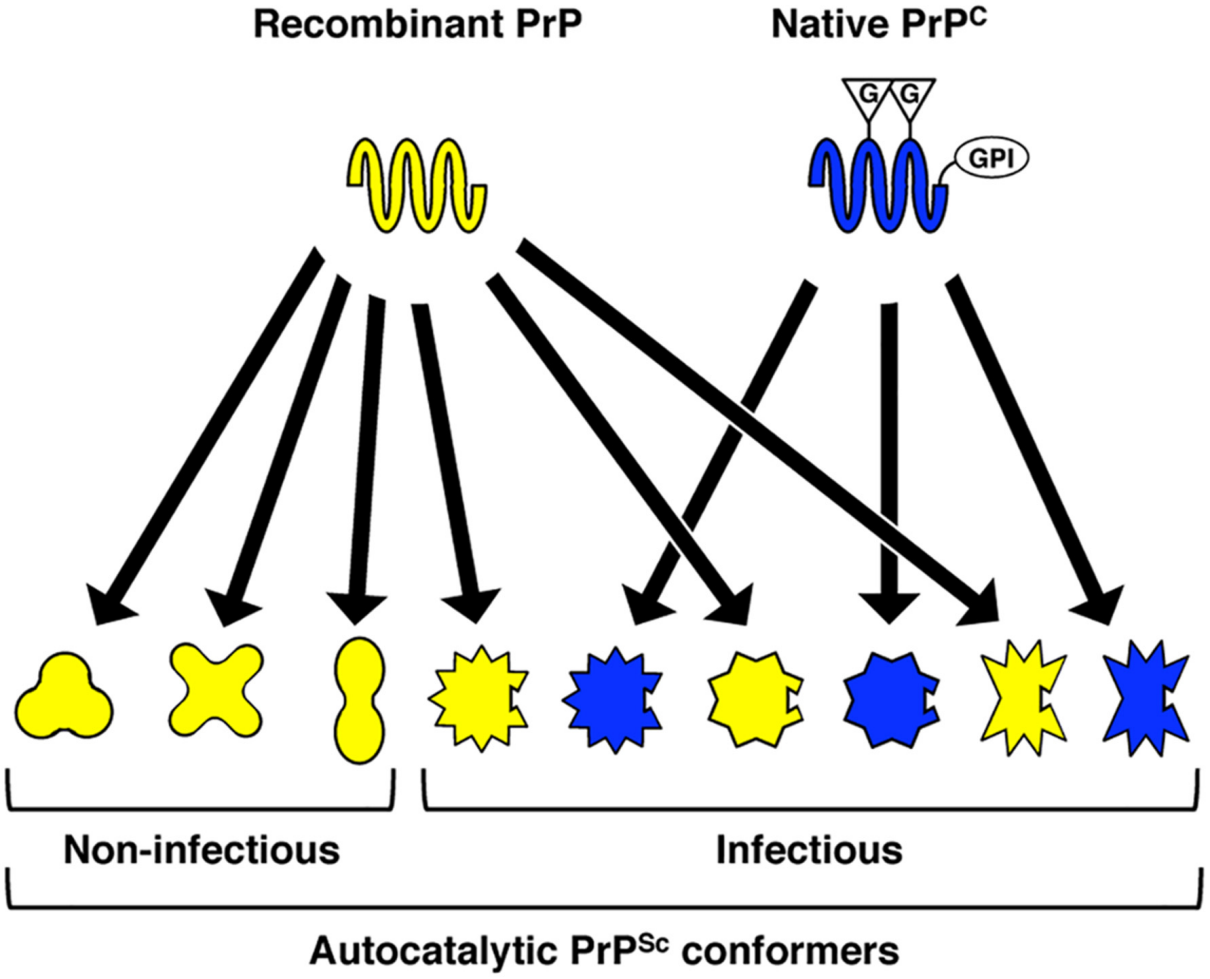
Proposed model, supported by structural and functional data in the present study, to explain variation in specific infectivity between different recombinant PrP^Sc^ conformers. Recombinant PrP, which lacks post-translational modifications, has fewer conformational constraints than native PrP^C^ and can adopt a variety of autocatalytic PrP^Sc^ conformations. Only those autocatalytic PrP^Sc^ conformers that can also structurally accommodate the post-translational modifications of native PrP^C^–in the case of the conformers studied presently, a substrate GPI anchor—are capable of biological infectivity. G = N-linked glycan; GPI = GPI anchor.

To integrate the results of our structural and functional comparison of cofactor and protein-only PrP^Sc^, we propose a model to account for the striking variation in specific infectivity observed for recombinant PrP^Sc^ conformers [9–15] (Fig 6). In this model, the PrP polypeptide backbone, represented by recombinant PrP, is capable of adopting a wide variety of autocatalytic conformations. However, post-translationally modified, native PrP^C^ can only adopt a subset of these conformations, and this subset represents the infectious recombinant PrP^Sc^ conformers. From previous studies of PrP and other proteins, there is evidence to suggest that post-translational modifications can alter or restrict protein folding pathways. Indeed, N-linked glycans are known broadly to have chaperone-like effects and to contribute to protein stability [51]. In the case of PrP, it has been observed that the presence of glycans can alter the rate of amyloid fibril formation [52,53], and that by restricting available PrP glycoforms it is possible to control prion strain susceptibility [54]. Whether GPI anchors play a role in protein folding or misfolding is less clear. The loss of the GPI anchor of PrP (which is concomitant with a significant reduction in PrP^C^ glycosylation *in vivo* [55]) appears to have only a modest effect on PrP^Sc^ structure [56], but substantially alters the biochemical properties of PrP^Sc^ and promotes the formation of fibrillar aggregates [57,58] and interferes with PrP^Sc^ replication *in vitro* [49]. Interestingly, the co-expresssion of anchorless and wild-type PrP^C^ molecules *in vivo* appears to enhance host susceptibility to recombinant PrP amyloid fibrils [59], while the experimental addition of a GPI anchor to the amyloidogenic yeast protein, Sup35p, prevents the formation of fibrillar structures, leading instead to the formation of PrP^Sc^-like, non-fibrillar aggregates [60]. While the data from the present study specifically point to a role for the GPI anchor of native mouse PrP^C^ in restricting the range of recombinant PrP^Sc^ conformers that possess infectious activity, we cannot exclude the possibility that N-linked glycans also influence the infectivity-associated recombinant PrP^Sc^ conformational space. In fact, we speculate that the boundaries of this subset of the conformational space are likely to be highly context-dependent, determined by a complex interplay between the polypeptide sequence of a given PrP^C^ substrate molecule and all of its associated post-translational modifications.

In conclusion, we report for the first time a structural and functional comparison between two autocatalytic recombinant PrP^Sc^ conformers that share the same origin and biochemical behavior, but differ >10^5^-fold in infectious titer for wild-type mice. Based on our findings, we suggest that those autocatalytic recombinant PrP^Sc^ conformers which are also highly infectious contain specific structural features in a limited number of PrP domains, and that these features may be required in order to accommodate specific substrate post-translational modifications during native PrP^C^ misfolding *in vivo*.

## Materials and Methods

### Preparation of cofactor and protein-only PrP^Sc^ by sPMCA

Cofactor and protein-only PrP^Sc^ [10]were generated by sPMCA as described [10]. Briefly, 100–200 ul reactions containing 6 μg/ml recombinant mouse PrP 23–230 (recombinant PrP) in conversion buffer [20 mM Tris (pH 7.5), 135 mM NaCl, 5 mM EDTA (pH 7.5), 0.15% Triton X-100] were supplemented with either brain-derived cofactor [10] for cofactor PrP^Sc^ propagation, or water for protein-only PrP^Sc^ propagation. Reactions were seeded with 1/10^th^ volume of converted cofactor or protein-only PrP^Sc^ PMCA product and sonicated with 15-s pulses every 30 min for 24 h at 37°C. After 24 h of PMCA, 1/10^th^ volume of the reaction was used to seed fresh substrate cocktail and the 24 h sonication program was repeated. PMCA reactions were sonicated in microplate horns using a Misonix S-4000 power supply (Qsonica) set to amplitude 50–60. Sample tubes were sealed with Parafilm (Bemis Company) and the sonicator horn was soaked in 100% bleach prior to switching to propagation of a different PrP^Sc^ species in order to prevent cross-contamination.

### Detection of cofactor and protein-only PrP^Sc^

Formation of cofactor and protein-only PrP^Sc^ was monitored by digestion of PMCA samples with proteinase K (PK) and Western blotting. Samples were treated with 25 μg/ ml PK (Roche) for 30 min at 37°C, and digestion reactions quenched by the addition of SDS-PAGE loading buffer and heating to 95°C for 10 min. SDS-PAGE and Western blotting were performed as described previously [10] using mAb 27/33, unless otherwise specified.

### Purification and epitope mapping of cofactor and protein-only PrP^Sc^ PK-resistant cores

All centrifugation was done at 4°C. Cofactor and protein-only PrP^Sc^ PMCA products were treated or mock-treated with PK as described above and the reaction quenched by the addition of PMSF to 5 mM final concentration. To remove PK, excess lipids, and any soluble recombinant PrP digestion products, digested PrP^Sc^ was washed twice with nOG wash buffer (1% n-octyl-beta-D-glucopyranoside (nOG), 150 mM NaCl, 8.3 mM Tris pH 7.2) by centrifugation at 100,000 rcf for 1 h, with resuspension by sonication (60-s pulse, 70 amplitude) followed by brief vortexing. After the second wash, samples were pelleted by centrifugation at 100,000 rcf for 1 hr and resuspended in conversion buffer by sonication and vortexing to a recombinant PrP concentration of 6 μg/ml for use in epitope mapping and sPMCA experiments.

Epitope mapping of the cofactor and protein-only PrP^Sc^ PK-resistant cores was performed using mAbs 6D11 [61] and R2 [62]. Aliquots of identical purified samples were run on SDS-PAGE, transferred to PVDF membrane, and processed independently by Western blotting using trays and containers that had never been in contact with anti-PrP antibodies.

### sPMCA with native PrP^C^ and deglycosylated PrP^C^ substrates

For normal brain homogenate sPMCA, brain homogenates were prepared at 10% (w/v) in conversion buffer (PBS containing 1% (v/v) Triton X-100 and cOmplete protease inhibitor cocktail (Roche)) from healthy C57BL/6 mice, as described by Castilla *et al*. [5]. Homogenates were clarified by brief sonication (F60 Sonic Dismembrator (Fisher Scientific), three 5-s pulses at ~1.8 amplitude), followed by centrifugation at 500 rcf for 15 min. Clarified substrates were then seeded with 1/10^th^ volume of purified PrP^Sc^ samples, and sPMCA carried out as described above, with 20-s sonication pulses every 30 min.

For deglycosylated PrP^C^ sPMCA, diglycosylated native PrP^C^ was first purified from normal mouse brain and then fully deglycosylated by treatment with PNGase F (New England Biolabs) as described [63]. Deglycosylated PrP^C^ sPMCA reactions [63] were seeded with 5% (v/v) of recombinant or brain-derived PrP^Sc^ and sonicated as described above for normal brain homogenate sPMCA.

### Deuterium exchange and quenching of cofactor and protein-only PrP^Sc^

All centrifugation was done at 4°C. Samples were prepared for deuterium exchange as described previously [32] with the following modifications. For each PrP^Sc^ species, converted PMCA cocktail was washed twice with nOG wash buffer (1% n-octyl-beta-D-glucopyranoside (Anatrace), 150 mM NaCl, 8.3 mM Tris pH 7.2) by centrifugation at 100,000 rcf for 1 h, with resuspension by 60 s of sonication at 70 amplitude followed by brief vortexing. After the second wash, samples were pelleted by centrifugation at 100,000 rcf for 1 hr and resuspended in a volume of mock labeling buffer (150 mM NaCl, 8.3 mM Tris, pH 7.2) by sonication and vortexing immediately prior to the initiation of deuterium exchange. Deuterium exchange was initiated by the addition an equal volume of labeling buffer (D_2_O containing 150 mM NaCl, 8.3 mM Tris, pH* 7.2, where pH* is the pH meter reading without taking into account the hydrogen isotope effect) and samples were incubated at room temperature (22°C). During the last 30 min of labeling, samples were pelleted by centrifugation at 100,000 rcf. Labeling buffer was removed and the samples were quenched on ice for 2 min with ice-cold quench buffer [0.8% formic acid, 6.4 M guanidine hydrocholride, 150 mM tris(2-carboxyethyl)phosphine (TCEP) (Pierce)]. Quenched samples were diluted with 3 volumes of ice-cold acid diluent (0.8% formic acid, 16.6% glycerol), transferred to chilled autosampler microvials, frozen on crushed dry ice, sealed and stored at -70°C until analysis. For all PrP samples, including those described below, Western blotting of quenched material was performed and confirmed the presence of 1–2 μg recombinant PrP per sample vial.

### Deuterium exchange and quenching of α-helical recombinant PrP and preparation of equilibrium-deuterated controls

Deuterium exchange and quenching of normally folded, **α**-helical recombinant PrP (α-PrP) was performed as described above for cofactor and protein-only PrP^Sc^, with the following modifications. Recombinant PrP was resuspended to 1.0 mg/ml in water and an equal volume of 2x labeling buffer (D_2_O containing 300 mM NaCl, 16.6 mM Tris, pH* 7.2) was added to initiate deuterium exchange. Thirty minutes prior to quenching, an aliquot of the labeling reaction was placed at 4°C to replicate the temperature change experienced by PrP^Sc^ samples during centrifugation. Deuterium-labeled α-PrP was then quenched on ice for 2 min by the addition of 1.25 volumes of ice-cold quench buffer containing 700 mM TCEP. To the quenched samples was added 1.30 volumes of ice-cold acid diluent prior to aliquoting into autosampler microvials and freezing on dry ice, as described above.

Equilibrium-deuterated samples were prepared by resuspension of recombinant PrP in 2.5 or 6.0 M guanidine hydrochloride solution containing a 1:1 molar ratio of protons:deuterons by mixing appropriate quantities of H_2_O, D_2_O, guanidine HCl and guanidine (D_6_) DCl (Cambridge Isotope Laboratories, Andover, MA). Deuterium exchange was allowed to proceed for 72 hours at room temperature (22°C) prior to quenching as described above for α-PrP.

### Quantification of co-sedimentation of protease-sensitive, insoluble PrP in DXMS PrP^Sc^ samples

To estimate the fraction of non-specifically aggregated PrP that could potentially co-sediment during the purification of PrP^Sc^ samples for DXMS, mock-seeded PMCA reactions were performed using PMCA cocktail supplemented with brain-derived cofactor or water. After 24 h of PMCA, the mock-seeded PMCA reactions were purified by ultracentrifugation as described above for DXMS samples and the fraction of the input PrP that was recovered as non-specifically aggregated, insoluble PrP was quantified by Western blot.

### Analysis of deuterium incorporation

Measurement of deuterium incorporation by LC-MS was performed as described previously [32], with the following modifications. Samples were loaded onto the in-line immobilized fungal protease XIII column at a rate of 60 μl/min, allowed to digest for 3 min, and then pushed onto the in-line immobilized pepsin column at a rate of 20 μl/min. Peptides were collected during pepsin digestion on a C18 trap column (Michrom MAGIC C18AQ, 0.2x2) preceding the C18 resolving column (Michrom MAGIC C18AQ, 0.2x50). All measurements were made on an Orbitrap Elite mass spectrometer (Thermo Fisher Scientific), and data was analyzed as described previously [32].

### Immunoprecipitation of cofactor and protein-only PrP^Sc^ with mAb 15B3

Rat anti-mouse IgM-conjugated Dynabeads (Life Technologies) were washed according to the manufacturer’s protocol and coated with mAb 15B3 (Prionics) at 5 μg per 10 μl beads with gentle mixing at room temperature for 2 h. Coated beads were washed three times to remove unbound antibody and stored at 4°C for no more than one week prior to use. Converted cofactor or protein-only PrP^Sc^ PMCA cocktail was washed twice with nOG wash buffer as described above for the preparation of samples for DXMS. After the second wash, samples were collected by centrifugation at 100,000 rcf for 1 hr at 4°C and the pellet was gently washed with Prionics homogenization buffer (Prionics). Samples were then centrifuged at 100,000 rcf for 10 min at 4°C and the supernatant discarded. The pellet was resuspended in Prionics homogenization buffer by sonication (30-s pulse, 70 amplitude) and vortexing to a final concentration of ~60 ng/μl PrP. For each PrP^Sc^ sample, ~250 ng PrP was added to 0.5 ml Prionics IP buffer (Prionics) and to this was added 10 μl of beads that were either coated with 15B3 or uncoated. Samples were allowed to interact with the beads at room temperature for 4 h with gentle mixing, followed by two washes with Prionics IP buffer and resuspension of the beads in 2x SDS-PAGE loading buffer. Samples were incubated at 95°C for 10 min, briefly centrifuged to concentrate the beads and the supernatant was collected for analysis by Western blot.

### Raman spectroscopy of cofactor and protein-only PrP^Sc^

Cofactor and protein-only PrP^Sc^ were generated by sPMCA supplemented with synthetic plasmalogen PE (Avanti Polar Lipids) as the sole cofactor, as described previously [64]. Converted PMCA cocktail was digested with PK as described above and quenched by the addition of PMSF (Sigma Aldrich) to 2 mM final concentration. Digested samples were washed twice with nOG wash buffer, as described above, and then twice with water to remove residual buffer components and detergent. Samples were then resuspended in water to a concentration of ~140 ng/μl PrP by vortexing and a 15-s sonication pulse. 10 μl of the resulting sample was spotted onto a glass slide and allowed to dry under a stream of nitrogen. Once dry, another 10 μl was spotted on top of the first and again allowed to dry under nitrogen. Spotted samples were scanned using a WITec CRM200 Raman confocal light microscope, equipped with a 100x lens and a 514 nm argon laser with 45 mW output. An f/4, 300 mm imaging spectrograph was employed with 2 exit ports and a 600 lines/mm grating, with a Peltier-cooled CCD, 1340 x 100 pixel format, and a 16-bit camera controller. The fiber optic connecting the microscope with the spectrograph was 50 μm in diameter. Spectra were acquired using an integration time of 8 s, with two hardware and two software accumulations per shot and a spectral resolution of 4 cm^-1^. Presented spectra are averages of 20–30 shots. In each figure, the baseline was adjusted to zero and data points were joined with a smoothed line in Microsoft Excel. Although spectral normalization was not possible, data were collected with the same instrument at the same time from highly concentrated films of protein, and it can be expected that intensity differences between samples originate in structural differences between conformers.

### Ethics statement

All experiments involving mice in this study were conducted in accordance with protocol supa.su.1 as reviewed and approved by Dartmouth College’s Institutional Animal Care and Use Committee, operating under the regulations/guidelines of the NIH Office of Laboratory Animal Welfare (assurance number A3259-01).

## Supporting Information

S1 Fig Epitope mapping of cofactor and protein-only PrP^Sc^ protease-resistant cores. Western blots of mock and PK-digested PrP^Sc^ samples using mAb’s 6D11 (epitope comprising residues 93–109, with 97–100 as the major determinant of binding [1]) and R2 (epitope comprising residues 224–230 [2], the extreme C-terminus of mature PrP) following purification to remove all soluble PrP digestion and/or degradation products.
(TIF)

S2 Fig Western blot of cofactor and protein-only PrP^Sc^ samples subjected to structural analysis by DXMS in Fig 2. By densitometry, 301C-seeded, ME7-seeded and OSU-seeded cofactor PrP^Sc^ and OSU-seeded protein-only PrP^Sc^ have PK-resistant conversion efficiencies of 99, 82, 96 and 77%, respectively. Samples were purified as described and analyzed by DXMS. The resulting solvent accessibility profiles are shown in Fig 2.
(TIF)

S3 Fig DXMS purification of mock-seeded PMCA reactions recovers minimal protease-sensitive, insoluble PrP. To estimate the quantity of non-specifically aggregated PrP that could potentially co-sediment during the purification of PrP^Sc^ samples for DXMS labeling, mock-seeded PMCA reactions were performed using PMCA cocktail supplemented with brain-derived cofactor or water. After 24 h of PMCA, the mock-seeded PMCA reactions were purified by ultracentrifugation as described, with proportional samples of the supernatant/pellet taken during each of the three 100,000 rcf purification spins and analyzed by Western blot (labeled S_1_, P_1_, S_2_, P_2_, S_3_, P_3_). Sample S_0_ denotes mock-seeded PMCA material after 24 h of intermittent sonication and prior to ultracentrifugation. PK digestion was performed on the input and final pellet samples (S_0_ and P_3_, respectively) to determine protease resistance. Densitometry reveals that ~8% of the mock-seeded sample becomes non-specifically aggregated in PMCA reactions containing brain-derived cofactor (bottom panel, sample P_3_ vs S_0_). No non-specifically aggregated PrP was detected in protein-only mock-seeded PMCA reactions (top panel, sample P_3_ vs S_0_).
(TIF)

S4 Fig Regional solvent accessibility of α-PrP. 104 peptides, including different peptide charge states, were recovered in two technical replicates and the average deuterium incorporation of overlapping peptides was used to determine regional solvent accessibility, as described. Regions of NMR-assigned α-helix and β-strand structure are indicated by green spirals and black arrows, respectively [3].
(TIF)

S5 Fig Regional solvent accessibility of an independent protein-only PrP^Sc^ sample. A sample of non-infectious PrP^Sc^ generated in a parallel sPMCA amplification to those samples analyzed in Fig 2 and S2 Fig was purified and subjected to hydrogen-deuterium exchange as described. Regional solvent accessibility was determined from 226 recovered peptides, including different peptide charge states, as described.
(TIF)

S6 Fig Kinetic curves of deuterium incorporation for individual PrP^Sc^-derived peptides (N-terminal to and including peptide aa160-168). 63 peptides, including different peptide charge states, were identified in all DXMS technical replicates (n = 3 for 301C-seeded cofactor PrP^Sc^, orange triangles; n = 3 for ME7-seeded cofactor PrP^Sc^, green squares; n = 3 for OSU-seeded cofactor PrP^Sc^, red circles; n = 2 for OSU-seeded protein-only PrP^Sc^, blue diamonds). Data points represent the mean fractional deuterium incorporation at a given labeling duration, with error bars representing the standard deviation. Data points were fit with a single exponential function constrained to pass through the origin but without constraint on plateau height (solid lines). For peptides in which a fit was not possible with the specified constraints, data points are connected with straight dotted lines. S6 Fig includes deuterium incorporation data for peptides N-terminal to and including the aa160-168 peptide.
(TIF)

S7 Fig Kinetic curves of deuterium incorporation for individual PrP^Sc^-derived peptides (C-terminal to peptide aa160-168). 63 peptides, including different peptide charge states, were identified in all DXMS technical replicates (n = 3 for 301C-seeded cofactor PrP^Sc^, orange triangles; n = 3 for ME7-seeded cofactor PrP^Sc^, green squares; n = 3 for OSU-seeded cofactor PrP^Sc^, red circles; n = 2 for OSU-seeded protein-only PrP^Sc^, blue diamonds). Data points represent the mean fractional deuterium incorporation at a given labeling duration, with error bars representing the standard deviation. Data points were fit with a single exponential function constrained to pass through the origin but without constraint on plateau height (solid lines). For peptides in which a fit was not possible with the specified constraints, data points are connected with straight dotted lines. S7 Fig includes peptides C-terminal to the aa160-168 peptide.
(TIF)

S8 Fig Raman spectroscopy of cofactor and protein-only PrPSc, focusing on spectral regions assigned to CNH groups and tyrosine. Raman shifts corresponding to the ν(CN) mode at ~3300 cm^-1^ and the ν(CN) and δ(CNH) modes in the Amide II region (~1530–1580 cm^-1^) are shown, as well as a ν(CH) mode assigned to the tyrosine ring (~33075 cm^-1^). The data presented spanning Raman shifts of 1500–1800 cm^-1^ is the same as that presented in Fig 4, with different spectral information highlighted.
(TIF)

S9 Fig Biological replicate of deglycosylated PrP^C^ sPMCA conversion using cofactor and protein-only PrP^Sc^ seeds. Western blot showing three round sPMCA reactions using partially purified and deglycosylated PrP^C^ as the substrate and seeded with protein-only PrP^Sc^, cofactor PrP^Sc^, or prion-infected brain homogenate, as indicated. This represents a biological replicate of the experiment shown in Fig 5. All samples shown are from an identical exposure/image of a single membrane, with irrelevant samples removed so that the experimental samples are adjacent to one another.
(TIF)

S1 References Supporting Information references. (DOCX)
