# Supplementary figures and images for: A Structural and Functional Comparison Between Infectious and Non-Infectious Autocatalytic Recombinant PrP Conformers
Source: PLoS Pathog. 2015 Jun 30;11(6):e1005017. doi: 10.1371/journal.ppat.1005017 (PMC4488359; doi:10.1371/journal.ppat.1005017)

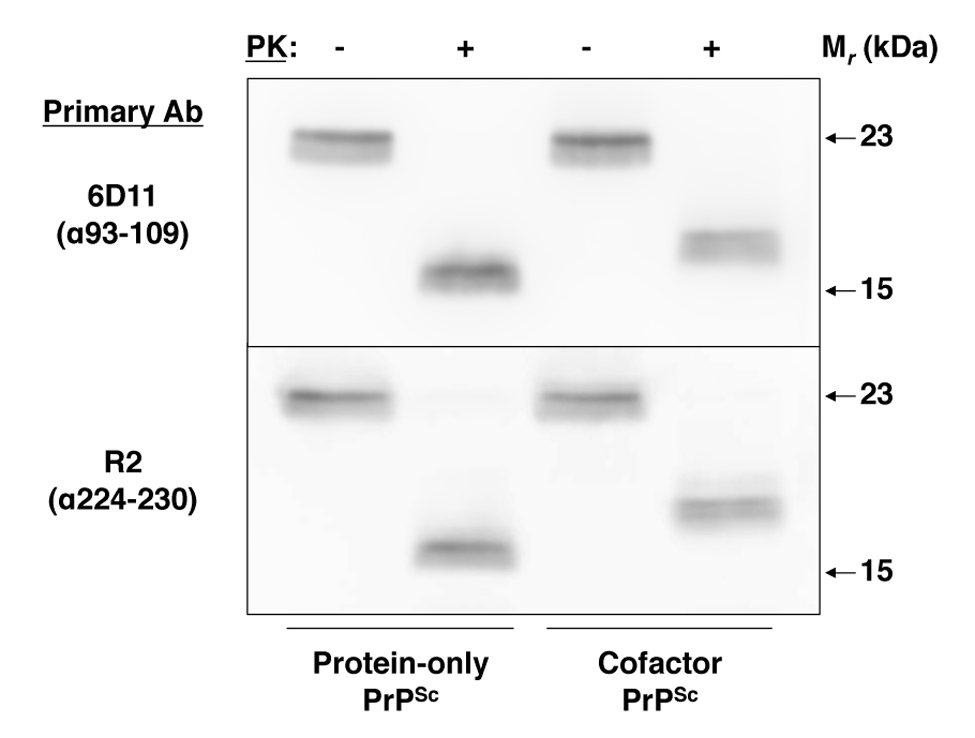

Supplement: S1 Fig — Western blots of mock and PK-digested PrPSc samples using mAb’s 6D11 (epitope comprising residues 93–109, with 97–100 as the major determinant of binding [1]) and R2 (epitope comprising residues 224–230 [2], the extreme C-terminus of mature PrP) following purification to remove all soluble PrP digestion and/or degradation products. (TIF) [file ppat.1005017.s001.tif]

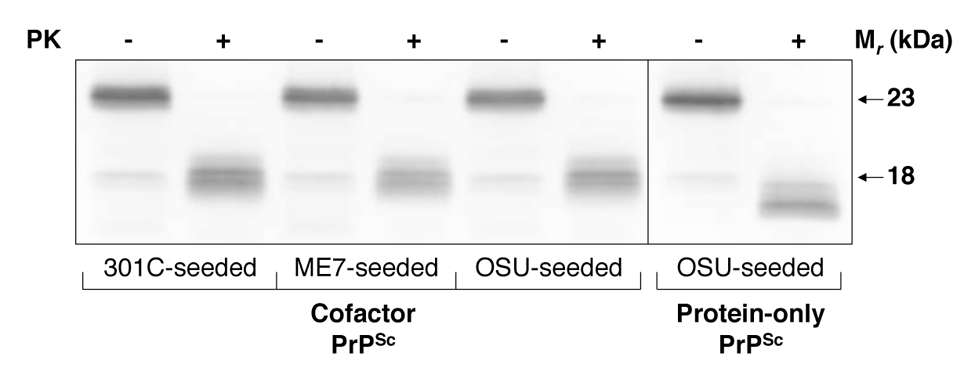

Supplement: S2 Fig — By densitometry, 301C-seeded, ME7-seeded and OSU-seeded cofactor PrPSc and OSU-seeded protein-only PrPSc have PK-resistant conversion efficiencies of 99, 82, 96 and 77%, respectively. Samples were purified as described and analyzed by DXMS. The resulting solvent accessibility profiles are shown in Fig 2. (TIF) [file ppat.1005017.s002.tif]

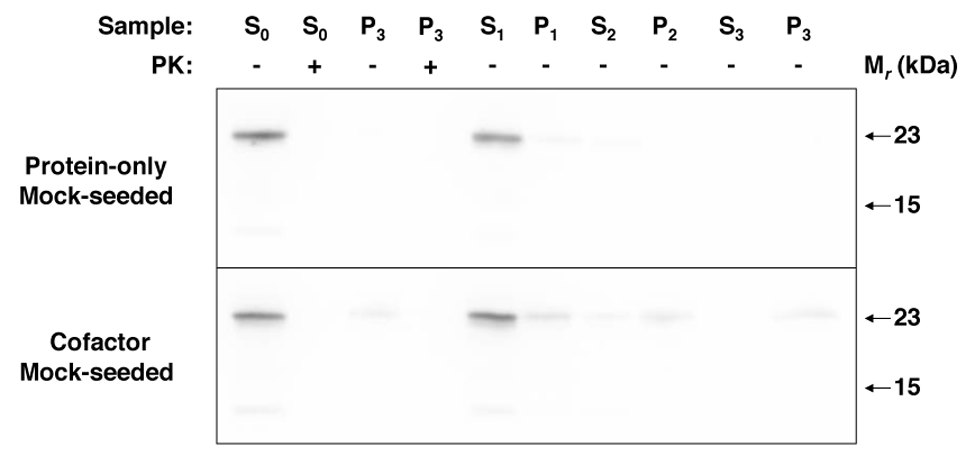

Supplement: S3 Fig — To estimate the quantity of non-specifically aggregated PrP that could potentially co-sediment during the purification of PrPSc samples for DXMS labeling, mock-seeded PMCA reactions were performed using PMCA cocktail supplemented with brain-derived cofactor or water. After 24 h of PMCA, the mock-seeded PMCA reactions were purified by ultracentrifugation as described, with proportional samples of the supernatant/pellet taken during each of the three 100,000 rcf purification spins and analyzed by Western blot (labeled S1, P1, S2, P2, S3, P3). Sample S0 denotes mock-seeded PMCA material after 24 h of intermittent sonication and prior to ultracentrifugation. PK digestion was performed on the input and final pellet samples (S0 and P3, respectively) to determine protease resistance. Densitometry reveals that ~8% of the mock-seeded sample becomes non-specifically aggregated in PMCA reactions containing brain-derived cofactor (bottom panel, sample P3 vs S0). No non-specifically aggregated PrP was detected in protein-only mock-seeded PMCA reactions (top panel, sample P3 vs S0). (TIF) [file ppat.1005017.s003.tif]

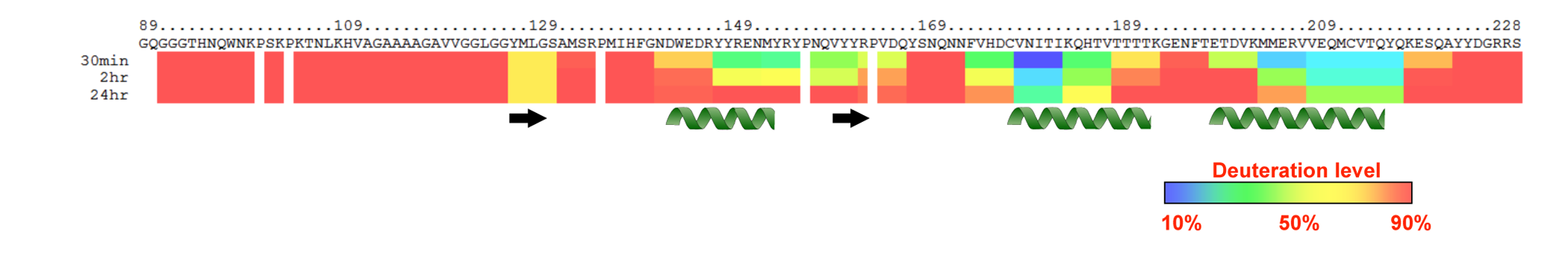

Supplement: S4 Fig — 104 peptides, including different peptide charge states, were recovered in two technical replicates and the average deuterium incorporation of overlapping peptides was used to determine regional solvent accessibility, as described. Regions of NMR-assigned α-helix and β-strand structure are indicated by green spirals and black arrows, respectively [3]. (TIF) [file ppat.1005017.s004.tif]

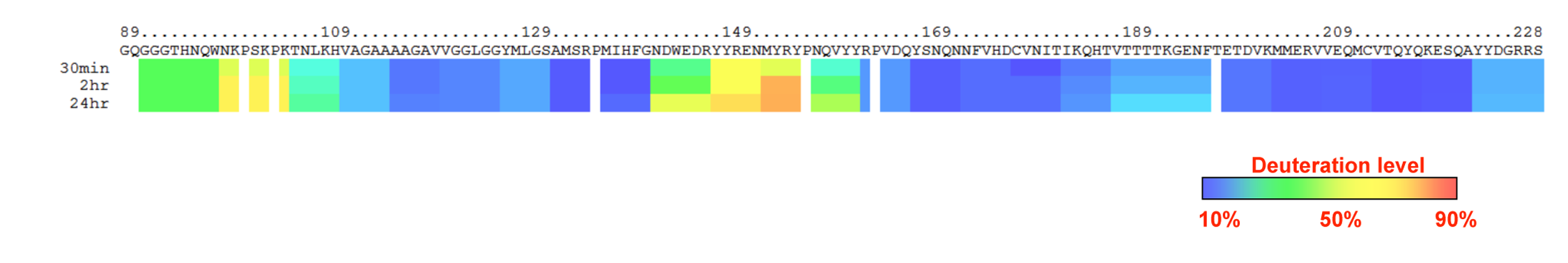

Supplement: S5 Fig — A sample of non-infectious PrPSc generated in a parallel sPMCA amplification to those samples analyzed in Fig 2 and S2 Fig was purified and subjected to hydrogen-deuterium exchange as described. Regional solvent accessibility was determined from 226 recovered peptides, including different peptide charge states, as described. (TIF) [file ppat.1005017.s005.tif]

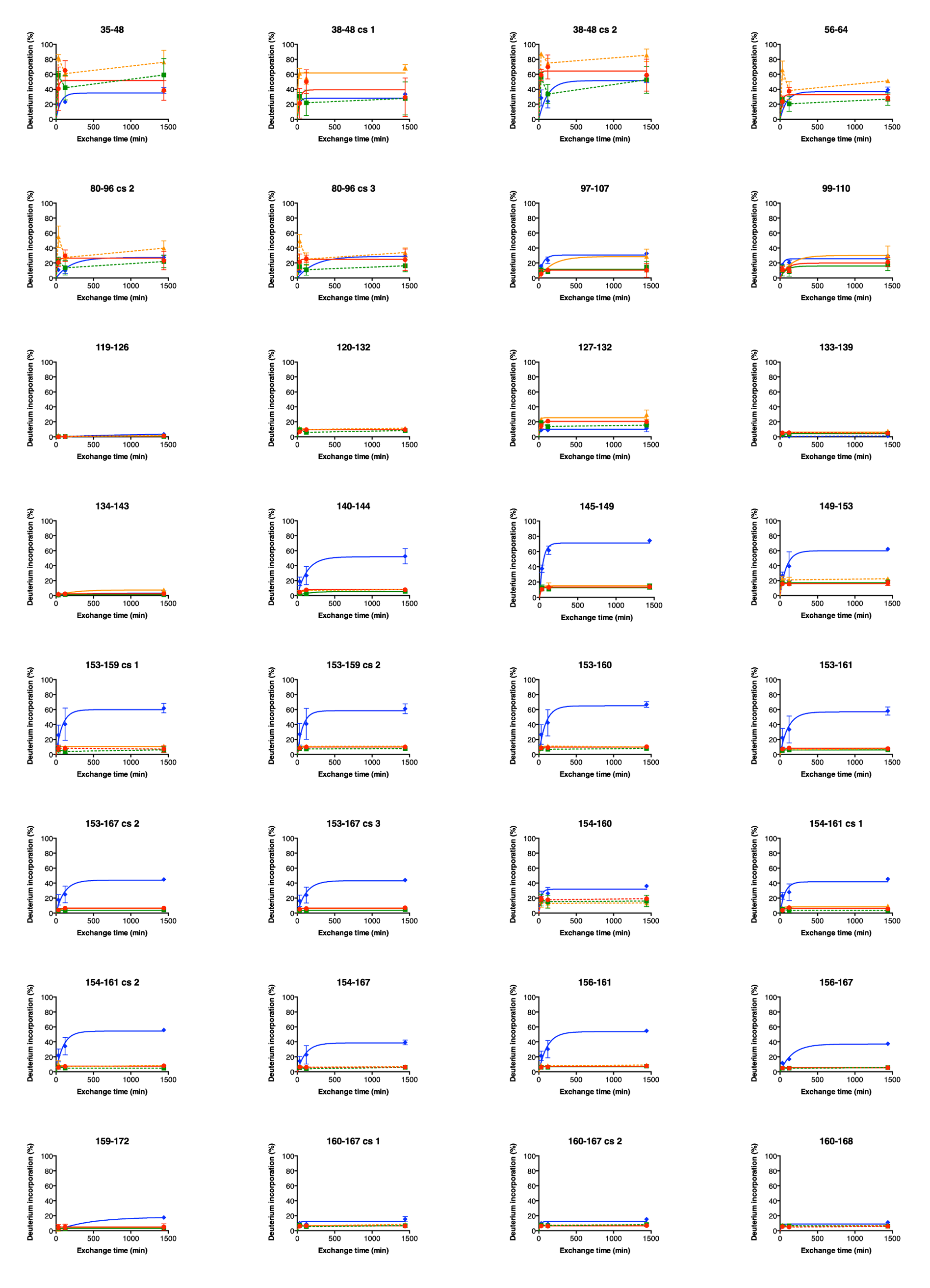

Supplement: S6 Fig — 63 peptides, including different peptide charge states, were identified in all DXMS technical replicates (n = 3 for 301C-seeded cofactor PrPSc, orange triangles; n = 3 for ME7-seeded cofactor PrPSc, green squares; n = 3 for OSU-seeded cofactor PrPSc, red circles; n = 2 for OSU-seeded protein-only PrPSc, blue diamonds). Data points represent the mean fractional deuterium incorporation at a given labeling duration, with error bars representing the standard deviation. Data points were fit with a single exponential function constrained to pass through the origin but without constraint on plateau height (solid lines). For peptides in which a fit was not possible with the specified constraints, data points are connected with straight dotted lines. S6 Fig includes deuterium incorporation data for peptides N-terminal to and including the aa160-168 peptide. (TIF) [file ppat.1005017.s006.tif]

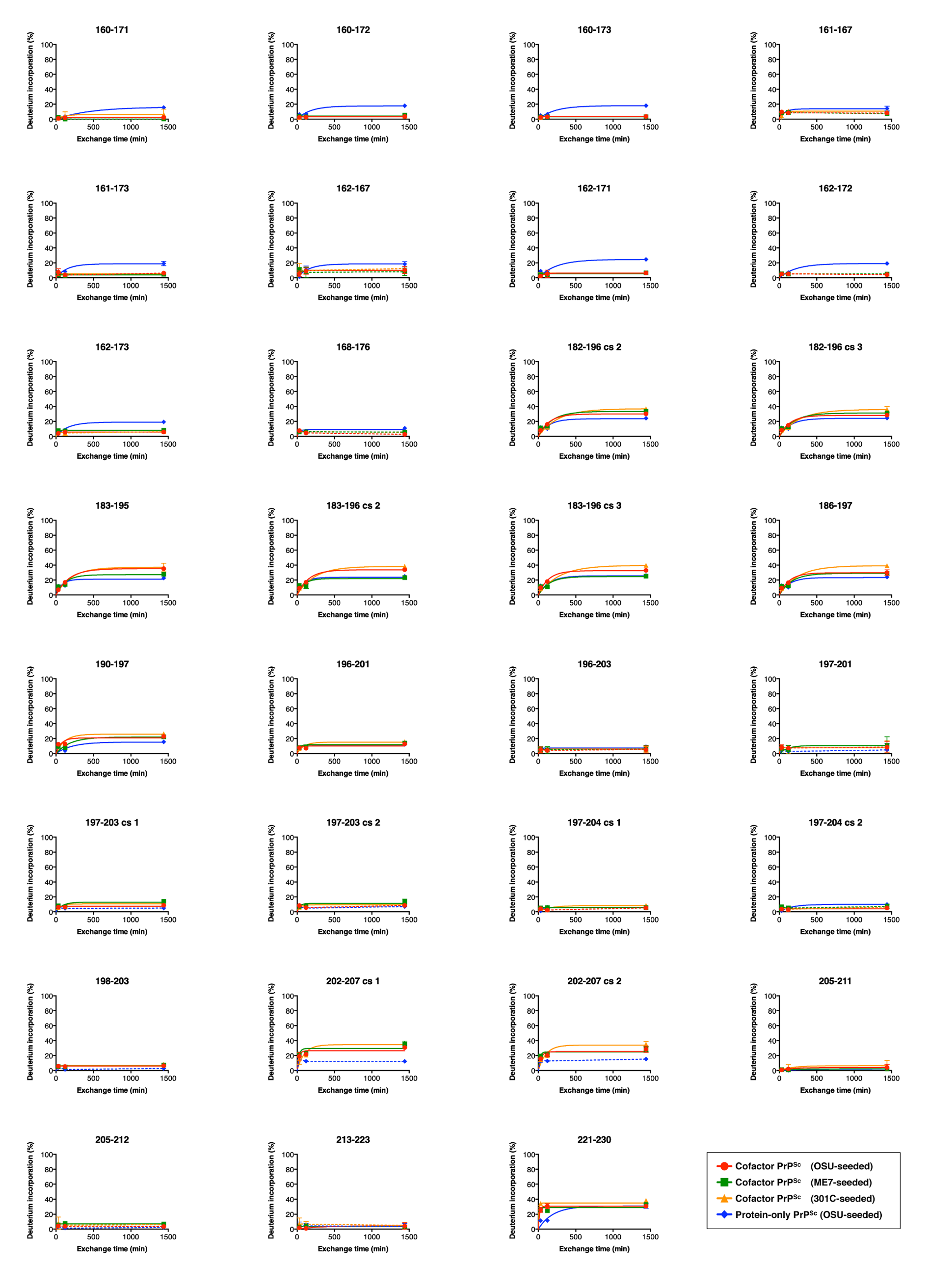

Supplement: S7 Fig — 63 peptides, including different peptide charge states, were identified in all DXMS technical replicates (n = 3 for 301C-seeded cofactor PrPSc, orange triangles; n = 3 for ME7-seeded cofactor PrPSc, green squares; n = 3 for OSU-seeded cofactor PrPSc, red circles; n = 2 for OSU-seeded protein-only PrPSc, blue diamonds). Data points represent the mean fractional deuterium incorporation at a given labeling duration, with error bars representing the standard deviation. Data points were fit with a single exponential function constrained to pass through the origin but without constraint on plateau height (solid lines). For peptides in which a fit was not possible with the specified constraints, data points are connected with straight dotted lines. S7 Fig includes peptides C-terminal to the aa160-168 peptide. (TIF) [file ppat.1005017.s007.tif]

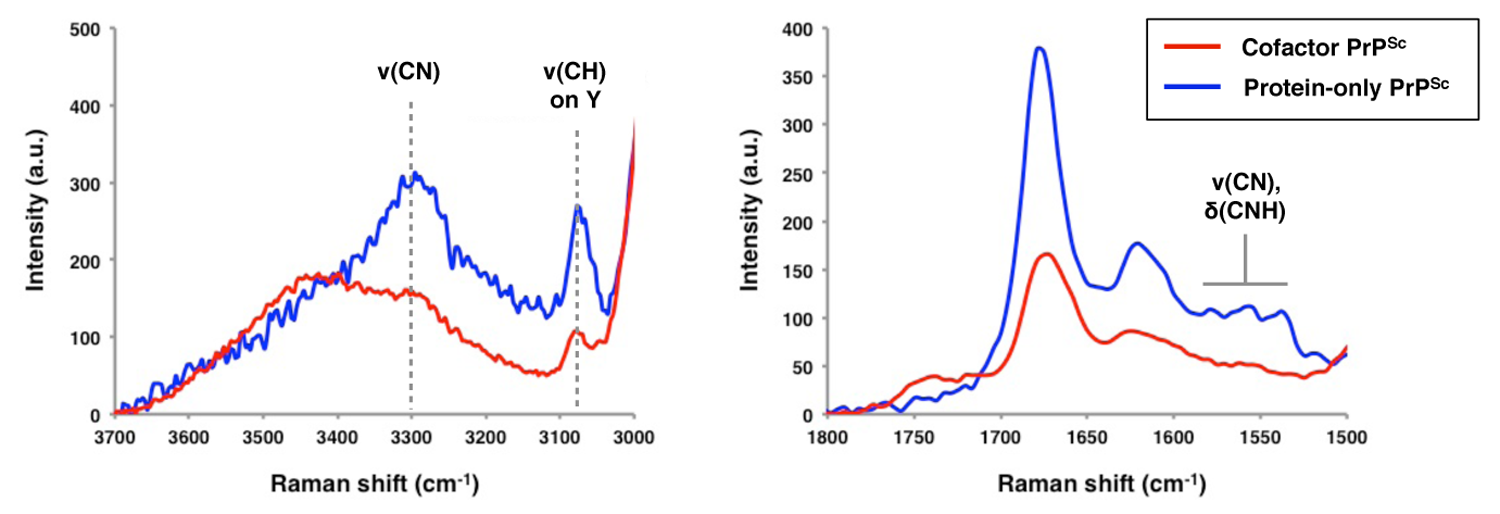

Supplement: S8 Fig — Raman shifts corresponding to the ν(CN) mode at ~3300 cm-1 and the ν(CN) and δ(CNH) modes in the Amide II region (~1530–1580 cm-1) are shown, as well as a ν(CH) mode assigned to the tyrosine ring (~33075 cm-1). The data presented spanning Raman shifts of 1500–1800 cm-1 is the same as that presented in Fig 4, with different spectral information highlighted. (TIF) [file ppat.1005017.s008.tif]

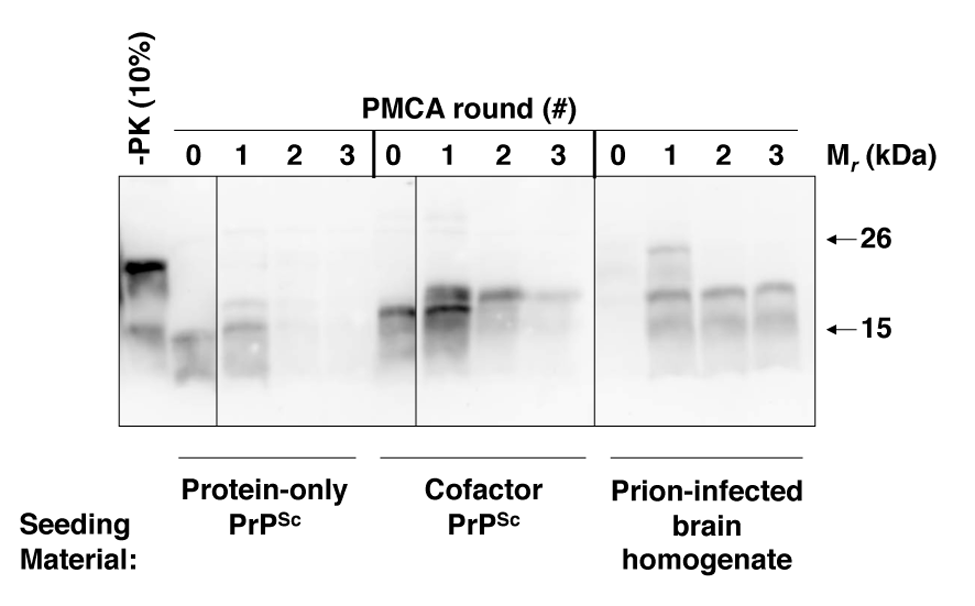

Supplement: S9 Fig — Western blot showing three round sPMCA reactions using partially purified and deglycosylated PrPC as the substrate and seeded with protein-only PrPSc, cofactor PrPSc, or prion-infected brain homogenate, as indicated. This represents a biological replicate of the experiment shown in Fig 5. All samples shown are from an identical exposure/image of a single membrane, with irrelevant samples removed so that the experimental samples are adjacent to one another. (TIF) [file ppat.1005017.s009.tif]
