# Supplementary material for: A Structural and Functional Comparison Between Infectious and Non-Infectious Autocatalytic Recombinant PrP Conformers
Source: PLoS Pathog. 2015 Jun 30;11(6):e1005017. doi: 10.1371/journal.ppat.1005017 (PMC4488359; doi:10.1371/journal.ppat.1005017)
Supplement: S1 References — (DOCX) [file ppat.1005017.s010.docx]

**Supporting Information References**

1. Spinner DS, Kascsak RB, Lafauci G, Meeker HC, Ye X, Flory MJ, et al. CpG oligodeoxynucleotide-enhanced humoral immune response and production of antibodies to prion protein PrPSc in mice immunized with 139A scrapie-associated fibrils. Journal of leukocyte biology. 2007;81(6):1374-85.

2. Williamson RA, Peretz D, Pinilla C, Ball H, Bastidas RB, Rozenshteyn R, et al. Mapping the prion protein using recombinant antibodies. J Virol. 1998;72(11):9413-8.

3. Riek R, Hornemann S, Wider G, Billeter M, Glockshuber R, Wuthrich K. NMR structure of the mouse prion protein domain PrP(121-321). Nature. 1996;382(6587):180-2.
